# Development of Notch-Free, Pre-Bent Rod Applicable for Posterior Corrective Surgery of Thoracolumbar/Lumbar Adolescent Idiopathic Scoliosis

**DOI:** 10.3390/jcm12175750

**Published:** 2023-09-04

**Authors:** Yoko Ishikawa, Satoshi Kanai, Katsuro Ura, Terufumi Kokabu, Katsuhisa Yamada, Yuichiro Abe, Hiroyuki Tachi, Hisataka Suzuki, Takashi Ohnishi, Tsutomu Endo, Daisuke Ukeba, Masahiko Takahata, Norimasa Iwasaki, Hideki Sudo

**Affiliations:** 1 Department of Orthopaedic Surgery, Hokkaido University Hospital, N15W7, Sapporo 060-8638, Hokkaido, Japan; y.ishikawa1105@gmail.com (Y.I.); notechoper@yahoo.co.jp (K.U.); m990015jp@yahoo.ne.jp (T.K.); yka2q@pop.med.hokudai.ac.jp (K.Y.); hitachi198885@gmail.com (H.T.); pingre_ya.4@icloud.com (H.S.); takashi.onishi.ortho@gmail.com (T.O.); m000053a@yahoo.co.jp (T.E.); daisuke922@nifty.com (D.U.); takamasa@med.hokudai.ac.jp (M.T.); niwasaki@med.hokudai.ac.jp (N.I.); 2 Department of Orthopaedic Surgery, Eniwa Hospital, 2-1-1 Kogane-Chuo, Eniwa 061-1449, Hokkaido, Japan; menchixp@gmail.com; 3 Division of Systems Science and Informatics, Hokkaido University Graduate School of Information Science and Technology, N14W9, Sapporo 060-0814, Hokkaido, Japan; 4 Department of Advanced Medicine for Spine and Spinal Cord Disorders, Faculty of Medicine, Graduate School of Medicine, Hokkaido University, N15W7, Sapporo 060-8638, Hokkaido, Japan

**Keywords:** adolescent idiopathic scoliosis, thoracolumbar/lumbar curve, pre-bent rod, iterative closest point method

## Abstract

Adolescent idiopathic scoliosis (AIS), the most common pediatric musculoskeletal disorder, causes a three-dimensional spine deformity. Lenke type 5 AIS is defined as a structural thoracolumbar/lumbar curve with nonstructural thoracic curves. Although a rod curvature will affect clinical outcomes, intraoperative contouring of the straight rod depends on the surgeon’s knowledge and experience. This study aimed to determine the optimum rod geometries to provide a pre-bent rod system for posterior spinal surgery in patients with Lenke type 5 AIS. These pre-bent rods will be beneficial for achieving proper postoperative outcomes without rod contouring based on surgeon experience. We investigated 20 rod geometries traced in posterior spinal reconstruction in patients with Lenke type 5 AIS. The differences between the center point clouds in each cluster were evaluated using the iterative closest point (ICP) method with modification. Before the evaluation using the ICP method, the point clouds were divided into four clusters based on the rod length using a hierarchical cluster analysis. Because the differences in the values derived from the ICP method were <5 mm for each length-based cluster, four representative rod shapes were generated from the length-based clusters. We identified four optimized rod shapes that will reduce operation time, leading to a decreased patient and surgeon burden.

## 1. Introduction

Adolescent idiopathic scoliosis (AIS) is a disorder that causes three-dimensional deformities of the pediatric spine [1,2]. Lenke et al. suggested a classification for AIS with six curve types, considering the lumbar spine modifier and thoracic kyphosis [3,4,5]. The type 5 curve is defined as a structural thoracolumbar/lumbar curve, with nonstructural upper-thoracic and main-thoracic curves [3,5,6].

The corrective surgery with an anterior approach for thoracolumbar/lumbar curves was developed by Dwyer et al. in the 1970s [7]. Although the anterior approach remains useful for Lenke type 5 AIS, posterior spinal fusion with pedicle screw instrumentation is currently the standard technique, with a relatively low complication rate [8,9,10]. Some authors have demonstrated that the posterior approach has no significant difference in the coronal and sagittal correction compared to the anterior approach, although the anterior approach has the advantage of saving fusion levels [11,12,13,14,15].

Although optimal rod contouring is essential for anatomical spinal correction, the rod contouring procedure highly depends on the surgeon’s knowledge or experience [16]. Additionally, the notches generated in rod contouring decrease the mechanical properties of the rod [17,18]. We previously developed anatomically designed notch-free, pre-bent rods for patients with Lenke type 1 or 2 AIS, which resulted in reduced intraoperative rod deformation and improved thoracic kyphosis after the correction [16,19]. However, this implantation system is not applicable to Lenke type 5 AIS. This study aimed to determine the optimum rod geometries to provide a pre-bent rod system for posterior spinal surgery in patients with Lenke type 5 AIS by classifying the rod shape before implantation.

## 2. Materials and Methods

### 2.1. Patients

After institutional review board approval (approval number: 020-0416), we included 20 consecutive patients with Lenke type 5 AIS (2 men and 18 women) who underwent posterior spinal fusion between 2021 and 2023 at our institutions. Informed consent for this study and the publication of the information were obtained from all the participants and their guardians or parents, as applicable. Patients with syndromic, congenital, and neuromuscular scoliosis were excluded. Patients with Lenke types 1–4 and type 6 AIS curves were also excluded. The average age and body height at operation were 14.7 ± 1.9 years (range, 12–18) and 157.7 ± 6.6 cm (range, 149–173), respectively.

### 2.2. Radiographic Parameters

We investigated multiple parameters using a preoperative and 2-week follow-up standing long-cassette posteroanterior, lateral radiographs, and computed tomography (CT) [19]. The coronal measurements included the main thoracic curve angle, the thoracolumbar and lumbar curve angles, and L4 tilt. The global coronal balance was evaluated using the distance between the C7 plumb line and the center sacrum vertical line (C7–CSVL). The sagittal measurement included the thoracic kyphosis (T5–12) and lumbar lordosis (L1–S1). The sagittal balance was evaluated in the interval between the C7 plumb line and the S1 posterior superior corner (sagittal vertical axis). The vertebral rotation was measured using the axial plane of the CT image. In addition, the rod angles outlined below were also measured as indicators of rod deformation.

### 2.3. Rod Angle

**Figure 1:**
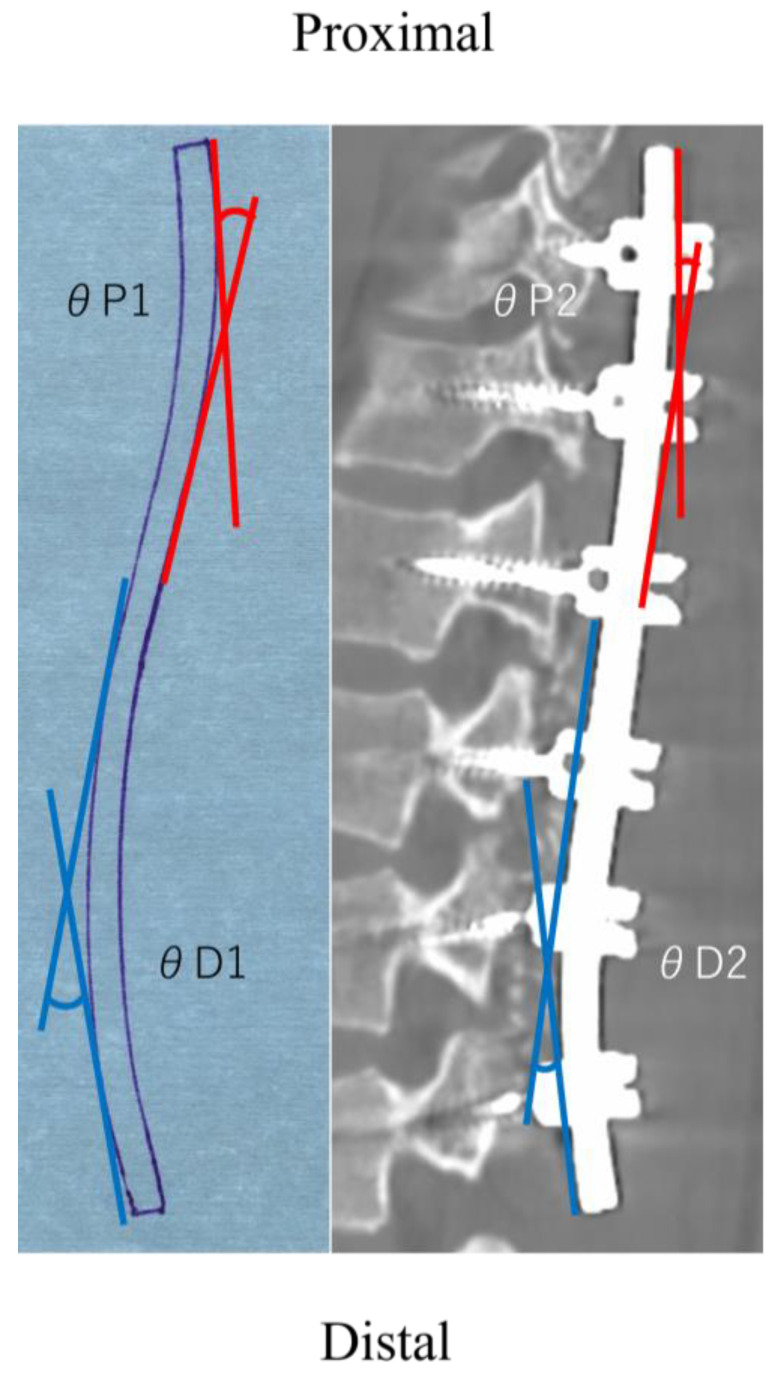
Definition of pre- and postoperative rod angles.

The rod angle was measured using the rod shape on the left side. Prior to applying the contouring rod to the screw head, the contours of the rod shapes were traced on paper [16]. The angle between the proximal and distal tangential lines was measured at the proximal and distal curvature before implantation (θP1 and θD1, respectively) (Figure 1). The postoperative implant rod shape was obtained from the Digital Imaging and Communications in Medicine (DICOM) data from the 1-week postoperative CT scan. DICOM data were used to reconstruct the sagittal rod images using a DICOM viewer software (OsiriX Lite 12.0.1, Pixmeo Labs, Geneva, Switzerland). The postoperative rod angles were evaluated in a similar manner to that of the preoperative measurements from the sagittal reconstructed rod images (θP2 and θD2, respectively) (Figure 1). The difference between θ1 and θ2 (θ1–θ2) was calculated as the rod deformation (Δθ) [20,21,22].

### 2.4. Surgical Techniques

The correction surgery was performed using 5.5 mm diameter cobalt–chrome alloy implant rods and polyaxial pedicle screws (Continuously Variable Simulation SPINAL SYSTEM, ROBERT REID INC., Tokyo, Japan). We avoided implantation to L4, L5, and S1 as the lowest instrumented vertebra (LIV), considering the postoperative degenerative changes in the remaining mobile segments. The operative procedures, in brief, were as follows [16,19]: After the posterior spinal elements were exposed, the placement of the pedicle screw was performed with the resection of all-level facets within the instrumentation level. Both side rods were contoured to achieve the ideal postoperative coronal and sagittal alignments. After both side rods were applied to all screw heads, both side rods were simultaneously rotated. An in situ rod-bending maneuver to add to the correction was not performed.

### 2.5. Algorithm for Analyzing and Identifying the Optimal Rod Shapes

The optimal shapes for the pre-cut and pre-bent rods were found after performing the following steps.

*Step 1: Generation of a center point cloud for existing rod shapes*

**Figure 2:**
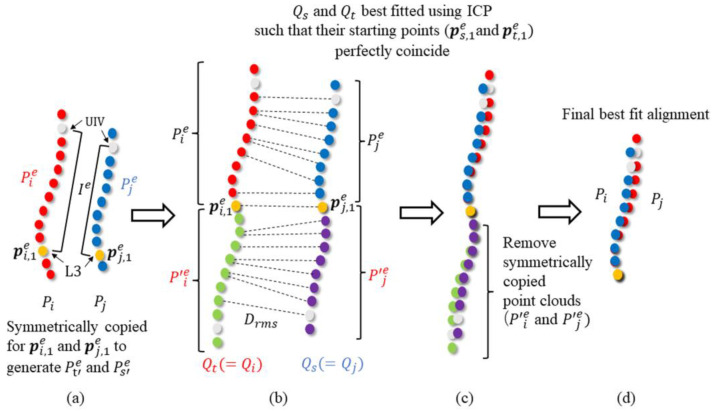
Evaluation of the difference between rods using the modified iterative closest point (ICP) method. (**a**) The center points of rods i and j that are included in the evaluation $interval\;I^{e}(from\;the\;UIV\;to\;L3)$ are selected as $P_{i}^{e}\;\mathrm{and}\;P_{j}^{e}$ from the original center point clouds $P_{i}\;and\;P_{j}$. The points closest to the fixation point of L3 in $P_{i}^{e}\;and\;P_{j}^{e}$ are selected as their starting points, $p_{i,1}^{e}$ and $p_{j,1}^{e}$, respectively. (**b**) The points $P_{i}^{e}\;and\;P_{j}^{e}$ are symmetrically copied with respect to their starting points $p_{i,1}^{e}$ and $p_{j,1}^{e}$. Then, $P_{i}^{e}\;and\;P_{j}^{e}$ and their symmetrically copied points ${P^{\prime }}_{i}^{e}\;and\;{P^{\prime }}_{j}^{e}$ are combined as $Q_{i}$ and $Q_{j}$. Of the two point clouds $Q_{i}$ and $Q_{j}$, the one with the longer length is selected as the target point cloud $Q_{t}$, and the other as the source point cloud $Q_{s}$. (**c**) The source point cloud $Q_{s}$ is best fitted to the target point cloud $Q_{t}$ using the ICP method. (**d**) The final best-fit alignment between point clouds $P_{i}\;and\;P_{j}$ was obtained by removing ${P^{\prime }}_{i}^{e}$ and ${P^{\prime }}_{j}^{e}$ from $Q_{s}$ and $Q_{t}$ at their best-fit position.

First, papers with hand-traced outlines of 20 rods were scanned and converted into a JPEG file. Next, a computer-aided design (CAD) operator manually fit a sequence of circular arcs and straight lines to the outline images of each rod shape on an AutoCAD 2016 (Autodesk, Inc., San Rafael, CA, USA) and Solidworks (Dassault Systèmes SolidWorks Corp, Waltham, MA, USA). Subsequently, the sequence of circular arcs and straight lines of the rods’ outlines were exported to an Excel file, and a center point cloud, $P_{i}$, of a rod i $\in R(R=\left\{1,2,\ldots ,20\right\}:$ a set of all rods), was generated by deriving the center curves of the input arcs and lines and by taking the constant-length sampling of the center curves using our original MATLAB (MATLAB R2022b for Windows: The Mathworks, Natick, MA, USA) code (Figure 2a).

*Step 2: Hierarchical cluster analysis for length-based grouping of the existing rods*

Since the curve lengths of the center point clouds of the 20 rods ranged from 145 to 220 mm, knowing which rods could be aggregated into one group based on length criteria was essential to prepare for the rod pre-cutting process. To this end, the difference in curve lengths between all the rods was evaluated as the distance between the different rods first, and a hierarchical cluster analysis with a complete linkage was conducted for the balanced, length-based grouping of the rods using our MATLAB code. The cluster analysis can identify the rod groups $G_{1},G_{2},{\ldots ,G}_{j},\ldots ,G_{K},(G_{j}\subseteq R,K:$ the total number of rod groups) such that the maximum difference in length among the rods in a group $G_{j}$ is less than the allowable value.

*Step 3: Evaluation of geometric difference among rods using a modified iterative closest point (ICP) method*

Because the initial positions and orientations of the center point clouds of the rods $\left\{P_{I}\right\}$ in rod group $G_{k}$ are not necessarily aligned, the center point clouds in $G_{k}$ were first best fitted to each other using our modified ICP [23] method before evaluating the difference in curve geometry among the rod shapes in $G_{k}$. As shown in Figure 2a, a subset of the center point clouds that were included only in the evaluation interval $I^{e}$ from the upper instrumented vertebra (UIV) to L3 were selected as targets of the alignment using the modified ICP, because L3 was fixed as the LIV. The center points included in the evaluation interval $I_{i}^{e}$ of a rod i were extracted from an original center point cloud I, which was defined as an evaluation point cloud $P_{i}^{e}$.

As shown in Figure 2b, in the point cloud alignment using the modified ICP, first, a point $I_{i,1}^{e}\left(\in P_{i}^{e}\right)$, closest to the fixation point of L3 was selected as the starting point for the evaluation of point cloud $P_{i}^{e}$. Then, all the other points in $P_{i}^{e}$ were point-symmetrically copied with respect to I, and the union of the original points $P_{i}^{e}$ and their copied points ${P^{\prime }}_{i}^{e}$ was created as the combined point cloud $Q_{i}(=P_{i}^{e}\cup {P^{\prime }}_{i}^{e})$. This copy and union process was performed for the evaluation of point cloud $P_{i}^{e}$ for all the rods.

As shown in Figure 2b, when aligning two combined point clouds $Q_{i}$ and $Q_{j}$ in a rod group $G_{k},$ the point cloud with the shorter evaluation interval length was selected as the source point cloud $Q_{s}$, and the point cloud with the longer interval length was chosen as target point cloud $Q_{t}$. Subsequently, the source point cloud $Q_{s}$ was best fitted to the target point cloud $Q_{t}$ using the following ICP method [23].

For the point cloud alignment using ICP, first, for all points $p_{s,m}$ in $Q_{s},$ the point $p_{t,c(m)}$ closest to $p_{s,m}$ is searched for in $Q_{t}$, where $c\left(m\right)$ denotes the index of the point in $Q_{t}$ that is closest to $p_{s,m}$ in $Q_{s}$. Next, the optimum position and orientation $\langle R^{\prime },t^{\prime }\rangle$ for $Q_{s}$ that best fits $Q_{s}$ to $Q_{t}$ can be found by solving the following Equations (1) and (2), where the mean square distance $D_{rms}^{2}$ between the closest point pairs $\left(p_{s,m},p_{t,c(m)}\right)$ is minimized [16].

(1) $$\langle R^{\prime },t^{\prime }\rangle =\arg\underset{\langle R,t\rangle }{min}\;D_{rms}^{2}$$

(2) $$D_{rms}=\sqrt{\frac{1}{\left|Q_{s}\right|}\sum_{p_{s,m}\in Q_{s}}{\left\|Rp_{s,m}+t-p_{t,c(m)}\right\|}^{2}}$$

where R denotes a 3×3 rotation matrix, t denotes a translation vector for transforming the source point cloud $Q_{S}$, and |$Q_{S}$| refers to the number of points in $Q_{S}$.

After that, every point $p_{s,m}$ in $Q_{S}$ is repositioned into its optimum position and orientation by applying $\langle R^{\prime },t^{\prime }\rangle$ to $p_{s,m}$ as defined in Equation (3):

(3) $$p_{s,m}\leftarrow R^{\prime }p_{s,m}+t^{\prime }$$

The derivation of the best-fit transformation for $Q_{S}$ using Equations (1) and (2) and the transformation of $Q_{S}$ using Equation (3) are repeated until the rotation and translation $\langle R^{\prime },t^{\prime }\rangle$ converge and, as a result, the final best-fit position and orientation of $Q_{S}$ to $Q_{t}$ is derived.

It is guaranteed that the centroids of $Q_{S}$ and $Q_{t}$ theoretically coincide in the best fit of $Q_{S}$ to $Q_{t}$ using the ICP, and the centroids of $Q_{S}$ and $Q_{t}$ are their starting points $p_{s,1}^{e}$ and $p_{t,1}^{e}$, respectively. Therefore, as shown in Figure 2c, the best-fit alignment of $Q_{S}$ and $Q_{t}$ can be obtained, such that both starting points $p_{s,1}^{e}$ and $p_{t,1}^{e}$ that are closest to the fixation points of L3 coincide with each other [23]. Finally, as shown in Figure 2d, the symmetrically copied points ${P^{\prime }}_{s}^{e}$ and ${P^{\prime }}_{t}^{e}$ are removed from $Q_{s}$ and $Q_{t}$ to obtain the final best-fit alignment of the center point clouds $P_{s}\;and\;P_{t}$ of two different rods s and t in a rod group.

*Step 4: Evaluation of rod shape difference*

If the maximum gap between one rod shape and the other is large, the created pre-bent rod may not be applied to the screw head during the corrective surgery. To this end, the maximum difference between rods i and j was evaluated as the maximum distance between their center point clouds, $P_{i}$ and $P_{j}$, under their best-fit aligned position as follows: The point clouds in a given a point cloud $P_{i}$ were first transformed into their best-fit position $P_{j}$ using the best-fit rotation $R^{*}$ and translation $t^{*}$ already derived from step 3. The maximum distance $D_{max}$ between point clouds $P_{i}$ and $P_{j}$ was evaluated as per Equation (4):

(4) $$D_{max}=\underset{p_{i,m}\in P_{i}}{\max}\left\{\left\|R^{*}p_{i,m}^{}+t^{*}-p_{j,d(m)}^{}\right\|\right\}$$

where $p_{j,d(m)}^{}$ is the point in $P_{j}$ that is closest to $R^{*}p_{i,m}^{}+t^{*}$.

This $D_{max}$ was used as an indicator of the difficulty of rod application during corrective surgery [16,23].

However, since $D_{rms}$ at the best-fitted alignment, as defined in Equation (2), represents the overall similarity in shape between center point clouds $P_{i}$ and $P_{j}$, the hierarchical cluster analysis in the following step was conducted using $D_{rms}$ as the distance to evaluate the similarity in shape between rods i and j in a rod group $G_{j}$.

*Step 5: Hierarchical cluster analysis among rod shapes*

Since the rod groups $G_{1},G_{2},{\ldots ,G}_{j},\ldots ,G_{K}$ were created based only on the similarity in rod length, various rod shapes might be included within a single group. Therefore, to assess the similarity in rod shapes in a rod group $G_{j}$, and identify the subgroups with similar rod shapes $H_{j1},H_{j2},\cdots ,H_{jL},(G_{j}={⋃}_{k\in \left[1,L\right]}H_{jk})$ in a given rod group $G_{j}$, a hierarchical cluster analysis was conducted using the criteria of complete linkage. $D_{rms}$ was adopted as the distance between two rod shapes in the cluster analysis [16,23]. The maximum allowable distance for $D_{rms}$ within a cluster of a subgroup $H_{jk}$ was determined as 5 mm according to a previous study [16]. The cluster analysis revealed a subgroup of rods with similar rod geometry and rod length.

*Step 6: Derivation of a pre-bent and pre-cut rod shape from the representative curve in rod subgroups*

Finally, for each subgroup of rods with a similar length and shape, $H_{jk}$, found in Step 5, a representative curve that best fits them was generated, and then a 3D model of the pre-bent and pre-cut rod shape was derived, whose center curve was identical to the representative curve.

Since all the center points of the rods $\left\{P_{i}|i\in H_{jk}\right\}$ were best fitted to each other in a subgroup $H_{jk}$ using ICP similar to that in step 3, a union of the best-fitted center points $P_{jk}^{U}={⋃}_{i\in H_{jk}}P_{i}$ was first created for the subgroup $H_{jk}$. Next, a smooth B-spline curve $C_{jk}^{U}$ was best fitted to all the center points included in $P_{jk}^{U}$ using the iterative least-square fitting method [16,23]. Because the best-fit curve $C_{jk}^{U}$ can be regarded as the curve representative of the center curve shapes of all the rods included in the subgroup $H_{jk}$, the curve $C_{jk}^{U}$ can be used as the center curve of the pre-bent and pre-cut rod shape for the rod subgroup $H_{jk}$ [16,23]. Therefore, the triangle mesh for a pre-bent and pre-cut rod shape was generated by sweeping a circle with a user-defined rod diameter along the B-spline curve $C_{jk}^{U}$ of the subgroup $H_{jk}$. Finally, the pre-bent and pre-cut rod shapes represented by the triangle meshes were saved as a standard triangulated language (STL) file.

## 3. Results

The patients’ demographic data are summarized in Table 1. Although the preoperative thoracolumbar/lumbar curve was 42.2°, the postoperative radiographs show an improvement to a thoracolumbar/lumbar curve of 5.9°. The sagittal plane analysis revealed that the preoperative lumbar lordosis was 46.4°, which increased significantly to 50.6° (*p* = 0.04). The preoperative and postoperative implant rod angles are listed in Table 2. The UIV was selected as T9 in seven patients, T10 in 11 patients, and T11 in two patients, whereas the LIV was L3 in all the patients. The proximal rod angle changed from an θP1 of 18.3° to an θP2 of 9.3°, and the distal rod angle changed from an θD1 of 30.8° to an θD2 of 15.9°, indicating that both the proximal and distal rod angles significantly decreased after the correction. There was no correlation between the change in rod angle and any of the radiographic parameters (Table 2).

**Figure 3:**
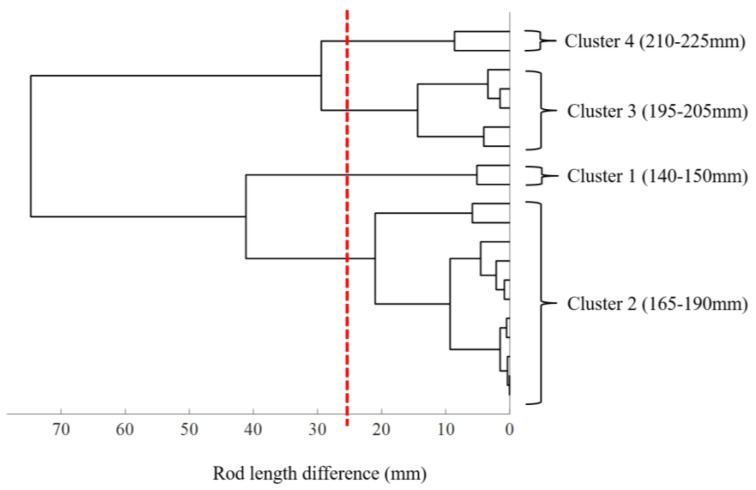
Hierarchical cluster analysis based on rod length.

**Figure 4:**
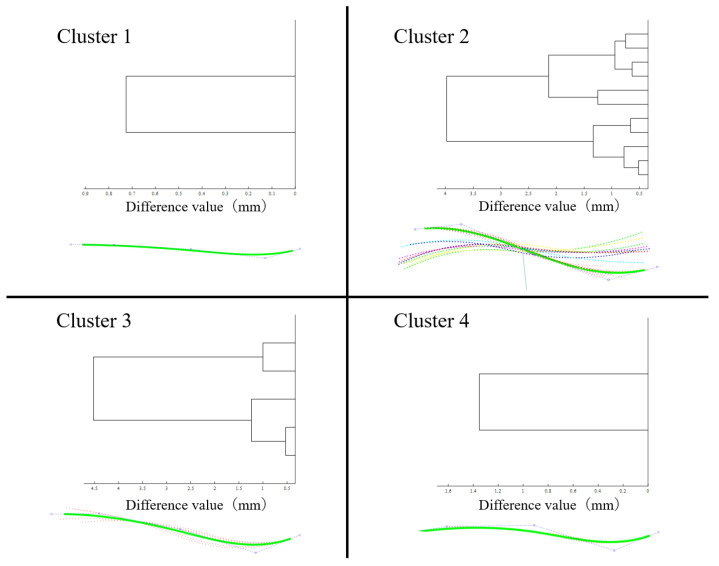
Dendrogram and best-fitted curves in each cluster obtained using the iterative closest point method.

**Figure 5:**
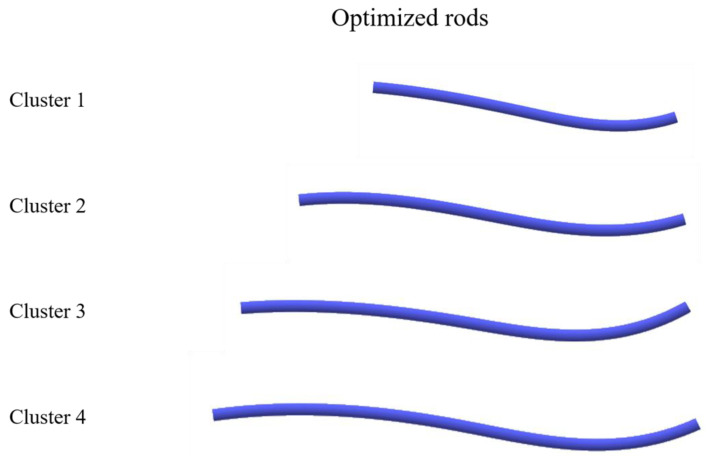
Standard triangulated language (STL) images of the optimized rod.

The rods were classified into four clusters according to their length (Figure 3). The number of rods was two in cluster 1 (140–150 mm), eleven in cluster 2 (165–190 mm), five in cluster 3 (195–205 mm), and two in cluster 4 (210–225 mm). The dendrogram obtained using the ICP method is shown in Figure 4. Without dividing the point clouds in the length-based cluster, the D_rms_, which is the overall difference between each point cloud, was <5 mm in all the clusters (Table 3). The D_rms_ and the D_max_ between the best-fitted curvature and the other point clouds of the rods in each cluster are shown in Table 3. The D_rms_ ranged from 0.21 to 1.91 mm, and the D_max_ ranged from 0.46 to 4.32 mm. Finally, the best-fitted curvature and STL images for the three-dimensional rods in each cluster are presented in Figure 5.

## 4. Discussion

The ICP method with modification was applied for identifying the optimal rod shape for the Lenke type 5 curve in this study. Our algorithm is modified at the point of best fitting between two rods at the target point, by making symmetrically copied points from the target point as compared to the ICP method used in previous studies [16,24], so that the fixation points on two rods that are the target points of the evaluation intervals are perfectly matched.

In the present study, in the cluster analysis for length classification, 20 rods were di-vided into four clusters with intervals of <25 mm. The D_rms_ was within 5 mm in each rod length-based cluster, indicating that it was possible for the point clouds for rod shape to converge to one best-fitted curve in each length-based cluster, because the thoracic pre-bent rod was created based on a D_rms_ < 5 mm in each cluster in a previous study [16]. Furthermore, the maximum D_rms_ and D_max_ between the best-fitted B-spline curvature and the other point clouds in each cluster were 1.9 and 4.7 mm, respectively, whereas the thoracic best-fitted curvature in a previous study had a D_rms_ of 2.2 mm and a D_max_ of 6.0 mm [16]. These thoracic pre-bent rods resulted in a good sagittal alignment in the correction of 27 patients with a Lenke type 1 curve without additional rod bending, suggesting that the four preset rod shapes with best-fitted B-spline curvature can be applied to the correction of patients with a Lenke type 5 curve without additional rod bending [19].

Considering mechanical implant failure and correction loss, the material and fatigue life of rods are also essential in developing a pre-bent rod [25]. Some authors [26,27,28,29,30] have described that cobalt–chromium alloy rods had a significantly higher stiffness than titanium alloy rods. In the current study, the rod angle of the convex side significantly decreased the proximal and distal curvature in the contoured rods, whereas the rod deformation did not affect the postoperative coronal and sagittal alignment parameters and changes. Although all the correction surgeries were performed using cobalt–chromium alloy rods, the titanium alloy rod can have a larger rod deformation that can influence postoperative outcomes. Furthermore, despite performing rod contouring prior to implantation in this series, the notch created by intraoperative bending should be avoided from the viewpoint of its impact on the postoperative coronal and sagittal outcomes due to rod deformation [31]. Notch-free cobalt–chromium alloy rods are optimum for the correction surgery for patients with Lenke type 5 curves to prevent rod deformation and obtain excellent radiographic parameters.

The twenty patients in this study showed improvements of 42.2° to 5.9° in the thoracolumbar/lumbar curve, 22.0° to 11.9° in the thoracic curve, and 20.7° to 6.7° in the L4 tilt. Additionally, thoracic kyphosis and lumbar lordosis were maintained as having good sagittal alignment. These postoperative outcomes will be promised in the correction surgery for Lenke type 5 AIS if using the presented four pre-bent rods. Moreover, these pre-bent rods not only provide good corrective outcomes but could also be useful in reducing the burden on patients and the surgeon in AIS correction. Although rod contouring depends on the surgeon’s experience or intuition, a mismatched rod configuration can lead to an incomplete correction and difficulty when applying the screws, which can increase anesthesia time and excessive bleeding. Some articles [32,33,34,35] have reported that using patient-specific pre-bent rods reduced the operating time for deformity corrections without rod contouring during surgery. The four preset rods will benefit patients and surgeons by shortening the operating time or by eliminating the dependence of the technique on the surgeon.

This study has some limitations. First, the four representative rod shapes for Lenke type 5 AIS were identified using traced rod shape data for 20 patients, meaning that the rod shape for Lenke type 5 was aggregated by one-fifth; however, it is unclear whether 20 cases are sufficient to create pre-bent rods for Lenke type 5 AIS correction. Nevertheless, clusters 1 and 2, with only two point clouds, would maintain the D_rms_ within 5 mm even if the number of point clouds in these clusters increased, because the D_rms_ of cluster 2, which has a maximum point cloud number of 11, was 4.0 mm. Second, these four rods can only be adopted for the correction of a thoracolumbar/lumbar curve with an LIV of L3, because L3 was selected as the LIV for all the cases in this study. We avoided selecting L4 or the vertebrae caudal to L4 as the LIV so as to not progress degenerative change by diminishing spinal mobile segments. However, it remains controversial whether to include L4 as the LIV in the correction of the thoracolumbar/lumbar curve, despite L4 being selected as the LIV by surgeons to prevent the risk of decompensation, especially for the large and rigid thoracolumbar/lumbar curve [36,37,38]. However, our algorithm used to develop the pre-bent rods is available to create the pre-bent rod for an LIV of L4 if there are rod shape data. Finally, the outcomes in the present study are based on 2-week follow-up radiographs and a 1-week-postoperative CT scan. Because the long-term clinical outcomes of the 20 patients in this study are unknown, the long-term outcomes of correction using these pre-bent rods should be validated. However, Yamada et al. [39] reported a good correction rate for Lenke type 5 posterior surgery, which was performed using the same surgical technique as in this study with manually bent rods, both immediately postoperative and 2 years after the operation.

## 5. Conclusions

We identified four optimum rod shapes (one-fifth of the total) from 20 patients to develop pre-bent rods designed for corrective surgery for thoracolumbar/lumbar adolescent idiopathic scoliosis. These pre-bent rods will be beneficial in achieving proper postoperative outcomes without rod contouring based on surgeon experience. They will also contribute to reducing the patients’ burden by diminishing operation time and blood loss.

## Figures and Tables

**Table 1:** Patients’ pre- and postoperative demographic data.

Radiographic Parameter	Pre-Operative	Postoperative	*p* Value
Thoracolumbar/lumbar curve (°)	42.2 ± 6.6	5.9 ± 2.4	<0.01
Thoracic curve (°)	22.0 ± 8.5	11.9 ± 8.0	<0.01
L4 tilt (°)	20.7 ± 4.3	6.7 ± 3.2	<0.01
Thoracic kyphosis (T5-12) (°)	24.9 ± 11.1	29.8 ± 8.0	0.02
Lumbar lordosis (L1-S1) (°)	46.4 ± 14.5	50.6 ± 12.2	0.04
C7 translation from CSVL (mm)	24.7 ± 14.6	16.9 ± 10.1	0.05
Apical vertebral translation (mm)	43.1 ± 9.3	8.9 ± 4.3	<0.01
Sagittal vertical axis (mm)	−0.4 ± 28.6	5.5 ± 25.2	0.34
Vertebral rotation (°)	20.3 ± 10.8	12.4 ± 5.0	<0.01
Proximal rod angle (°)	18.3 ± 6.7	9.3 ± 3.3	<0.01
Distal rod angle (°)	30.8 ± 8.0	15.9 ± 4.6	<0.01

**Table 2:** Correlation between rod deformation and radiographic parameters.

Variable	Rod Deformation (ΔθP)		Rod Deformation (ΔθD)	
	Correlation Coefficient	Statistical Significance	Correlation Coefficient	Statistical Significance
Postoperative main Cobb angle	*r* = 0.07	*p* = 0.76	*r* = 0.29	*p* = 0.18
Change in main Cobb angle	*r* = −0.20	*p* = 0.37	*r* = 0.01	*p* = 0.96
Postoperative L4 tilt	*r* = 0.03	*p* = 0.88	*r* = 0.25	*p* = 0.25
Change in L4 tilt	*r* = −0.10	*p* = 0.65	*r* = 0.11	*p* = 0.61
Postoperative lumbar lordosis	*r* = 0.21	*p* = 0.35	*r* = 0.01	*p* = 0.95
Change in lumbar lordosis	*r* = 0.30	*p* = 0.17	*r* = −0.36	*p* = 0.10
Postoperative thoracic kyphosis	*r* = 0.15	*p* = 0.50	*r* = 0.18	*p* = 0.43
Change in thoracic kyphosis	*r* = 0.22	*p* = 0.32	*r* = −0.23	*p* = 0.31

**Table 3:** The value of D_rms_ and D_max_ in each comparison.

	The Value between Each Point Cloud		The Value between Best-Fitted Curvature and the Other Point Clouds	
	D_rms_	D_max_	D_rms_	D_max_
Cluster 1	0.72	0.99	0.21	0.46
Cluster 2	3.98	8.35	1.18	4.32
Cluster 3	4.52	8.78	1.91	4.67
Cluster 4	1.35	2.19	0.57	1.16

## Data Availability

The data that support the findings of this study are available from the corresponding author on reasonable request.
